# PyGenePlexus: a Python package for gene discovery using network-based machine learning

**DOI:** 10.1093/bioinformatics/btad064

**Published:** 2023-01-31

**Authors:** Christopher A Mancuso, Renming Liu, Arjun Krishnan

**Affiliations:** Department of Computational Mathematics, Science and Engineering, Michigan State University, East Lansing, MI 48824, USA; Department of Biostatistics and Informatics, Colorado School of Public Health, University of Colorado-Denver Anschutz Medical Campus, Aurora, CO 80045, USA; Department of Computational Mathematics, Science and Engineering, Michigan State University, East Lansing, MI 48824, USA; Department of Computational Mathematics, Science and Engineering, Michigan State University, East Lansing, MI 48824, USA; Department of Biomedical Informatics, University of Colorado-Denver Anschutz Medical Campus, Aurora, CO 80045, USA

## Abstract

**Summary:**

*PyGenePlexus* is a Python package that enables a user to gain insight into any gene set of interest through a molecular interaction network informed supervised machine learning model. *PyGenePlexus* provides predictions of how associated every gene in the network is to the input gene set, offers interpretability by comparing the model trained on the input gene set to models trained on thousands of known gene sets, and returns the network connectivity of the top predicted genes.

**Availability and implementation:**

https://pypi.org/project/geneplexus/ and https://github.com/krishnanlab/PyGenePlexus.

**Supplementary information:**

Supplementary data are available at *Bioinformatics* online.

## 1 Introduction

Most functions, phenotypes and diseases are orchestrated by the complex interactions of many genes. To probe these biological contexts, researchers routinely generate sets of genes specific to those contexts using high-throughput, high-coverage technologies (Heller, 2002; Wang *et al.*, 2009). Additionally, numerous publicly available databases contain curated gene sets pertaining to various processes (Ashburner *et al.*, 2000; The Gene Ontology Consortium, 2019), diseases (Piñero *et al.*, 2015, 2017; Schriml *et al.*, 2019) and traits (Choobdar *et al.*, 2019). However, these gene sets are often incomplete, noisy and provide no information on how the genes in the set interact with each other, making it hard to fully understand the underlying biology that connects the genes. Hence, developing computational approaches that can provide insights into gene sets is a grand challenge in biomedical research (Jiang *et al.*, 2016; Piro and Cunto, 2012; Yang *et al.*, 2011).

**Fig. 1.**
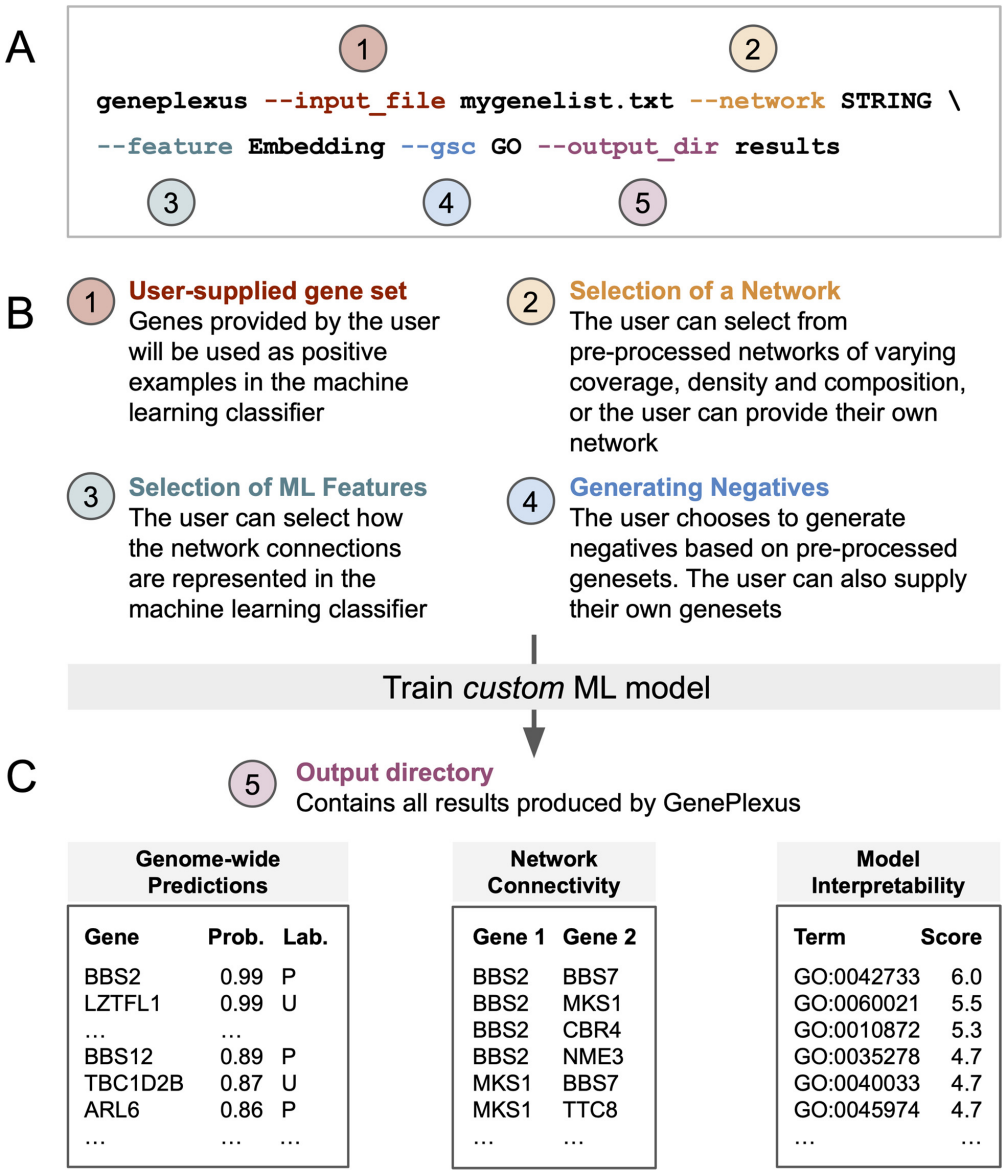
Running *PyGenePlexus* on the command line. (**A**) The *GenePlexus* model can be run with one simple command that (**B**) allows the user to select a number of different parameters and (**C**) obtain the results that are conveniently saved to the specified directory

Computational methods that incorporate information from genome-wide, context-specific molecular networks have recently shown state-of-the-art results in the task of prioritizing genes of interest and predicting other novel genes that may be highly related to the original gene set (Greene *et al.*, 2015; Köhler *et al.*, 2008; Krishnan *et al.*, 2016; Warde-Farley *et al.*, 2010). In a previous work, we have shown that using a supervised machine learning (ML) model that uses the connections from a genome-wide molecular network as the features in the ML model (referred to as *GenePlexus*) is a robust, data-driven way to computationally predict how associated a new gene is to a given input gene set (Liu *et al.*, 2020). *GenePlexus* produces more accurate gene classification performance compared to widely-used label propagation-based methods on diverse sets of tasks including functional, disease and trait predictions. In this work, we present *PyGenePlexus*, a python package that enables users to easily run the *GenePlexus* method on their input gene sets of choice on the command line (Fig. 1A).

## 2 Package overview

*PyGenePlexus* allows a user to input a set of genes and choose their desired network and its representation. *PyGenePlexus* then trains a custom ML model and returns the probability of how associated every gene in the network is to the user-supplied gene set, along with the network connectivity of the top predicted genes. Additionally, the software provides an interpretation of the custom model by comparing it to thousands of models previously trained on gene sets from known biological processes and diseases. The following sections describe the different parts of the package.

### 2.1 Downloading or supplying data

The *GenePlexus* method can utilize pre-processed information from genome-wide molecular networks and gene set collections from the Gene Ontology (GO) and DisGeNet. These data are archived on Zenodo (https://zenodo.org/record/6383205) and *PyGenePlexus* will automatically download the necessary data given the user input selections. Users can also supply their own networks and gene set collections to *PyGenePlexus*.

## 2.2 Inputs

The user must first provide a set of human genes, with valid ID types being Entrez, Symbols, Ensembl genes or Ensembl proteins (Fig. 1B). The user then chooses which molecular network to use and how that network should be represented in the ML model: as an adjacency matrix, an influence matrix, or a low-dimensional embedding of the network using node2vec (Grover and Leskovec, 2016; Liu and Krishnan, 2021). Finally, negatives are considered to be any gene annotated to at least one term in a user-chosen gene set collection (GO or DisGeNet), unless the gene is annotated to a term that is sufficiently ‘close’ to the user’s set. The set of positive and negative genes are then used to train a logistic regression binary classification model.

## 2.3 Results

*PyGenePlexus* returns the following results (Fig. 1C):

1. A prediction of how associated every gene in the network is to the input gene set.
2. The similarity of the model trained on the user-supplied gene set to thousands of models trained on gene sets from known pathways, processes and diseases.
3. The network connectivity of the top predicted genes.
4. The performance of the model through *k*-fold cross-validation.

For more information on the pre-processed data, input choices or results, see the package documentation.

## 3 Example use case

The biological insights achievable using *PyGenePlexus* can be illustrated by considering the genes associated with *Bardet-Biedl syndrome 1* (BBS1) in the DisGeNet database (Supplementary File S1). The example below utilizes ‘BioGRID’ as the network, ‘embeddings’ as the feature representation, and ‘DisGeNet’ as the background for selecting negative genes. Examining the genome-wide predictions from *PyGenePlexus* (Supplementary Table S1) shows that the gene *LZTFL1* (*leucine zipper transcription factor like 1*) at rank 2 was not in the original list of genes associated with the syndrome, and there is evidence that *LZTFL1* has a role in BBS1 (Marion *et al.*, 2012). Comparison of the model trained on BBS1 genes to models trained on known disease gene sets (Supplementary Table S2) shows that BBS1 model is highly similar to *Meckel syndrome* (both 8 and 1), which is a disease closely related to BBS1 (Forsythe and Beales, 2013; Karmous-Benailly *et al.*, 2005). Comparison of the BBS1 model to models trained on gene sets from known biological process shows that the top 10 results are terms relating to polydactylism, cholesterol and glycoside processes, and retina homeostasis, which relate to manifestations of BBS1 such as blindness, obesity and having extra fingers or toes (Forsythe and Beales, 2013) (Supplementary Table S3).

## 4 Discussion

*PyGenePlexus* is designed to be used by any researcher who wishes to gain insight about a gene set of interest using biological networks. To help accomplish this, we provide extensive documentation of the package (https://pygeneplexus.readthedocs.io/en/main/). Additionally, *PyGenePlexus* can be run in two ways: *pythonically* through the class-based method, or through a command line interface. Interacting directly with the Python code allows the user the ability to access all the functionalities of the package. The command line interface provides users who may not be familiar with Python an easier way to run the *PyGenePlexus* pipeline.

The *GenePlexus* method is also available through a well-documented, interactive web-server (https://www.geneplexus.net/). *PyGenePlexus* offers some complementary functionalities not available on the web-server. First, *PyGenePlexus* allows a user to provide their own networks and gene set collections, which can be tailored to better fit the context in which their gene set was generated (e.g. through the use of tissue-specific gene interaction networks). Second, the local installation of *PyGenePlexus* allows a user to allocate any computational resources they have at hand to repeatedly run the pipeline, for example to predict on many gene sets or iterate through all the network-feature combinations on a given gene set. Thus, *PyGenePlexus* is a powerful, intuitive, well-documented tool that is designed to be used by researchers with varying levels of programming ability, allowing users to gain network-based biological insights into their gene sets of interest.

## Supplementary Material

btad064_Supplementary_Data
